# Root exudates of alkaloids and flavonoids from *Lupinus angustifolius* plants

**DOI:** 10.1080/15592324.2026.2760037

**Published:** 2026-11-05

**Authors:** Hawar Halshoy, Shwana Ahmed Braim, Jawameer R. Hama

**Affiliations:** a Horticulture and Landscape Design Department, College of Agricultural Engineering Sciences, University of Sulaimani, Sulaymaniyah, Kurdistan Region, Iraq; b Chemistry and Forensics Department, School of Science and Technology, Nottingham Trent University, Nottingham, UK; c Department of Agroecology, Aarhus University, Slagelse, Denmark

**Keywords:** Root exudation dynamics, chemical ecology, allelochemicals, rhizosphere processes, plant metabolites, *Lupinus*

## Abstract

Root system traits play a crucial role in addressing current agricultural and environmental challenges. Because root dynamics and exudation are complex, researchers have used various approaches to investigate these processes. This study aims to assess Lupins (*Lupinus angustifolius* L.) plant biomass and test the effects of different collection media and collection duration on root exudation composition, focusing on alkaloids (13-hydroxylupanine, gramine, lupinine, lupanine, and sparteine) and flavonoids (apigenin, genistein, luteone, and rutin). The experiment included exudate collection intervals of 3–72 h and covered dynamic changes from 10 to 110 d after sowing (DAS). Deionized water and CaCl_2_ treatments were used as collection media to collect both alkaloids and flavonoids. The results revealed that fresh root weight reached its maximum at 90 DAS, while root dry weight and root length peaked at the final interval of 110 DAS. Root exudate collection from 20-day-old lupin roots using deionized water and CaCl_2_ as collection solutions indicated that deionized water facilitated the highest exudation of both alkaloids and flavonoids. Furthermore, exudate collection over 24 h with deionized water yielded the highest exudation of both alkaloids and flavonoids. Alkaloid exudation was significantly influenced throughout the growing season from 20 to 110 DAS. Similarly, flavonoid exudation fluctuated over time, with the highest levels recorded during the final growth stages. These findings provide insights into the dynamics of root exudation and may improve understanding of plant–soil interactions, secondary metabolite release, and adaptation in lupin and other species.

## Introduction

Plants have evolved complex processes to perceive and respond to their environment.^1^ Various biotic and abiotic factors, such as soil composition, nutrient availability, microbial interactions, temperature variations, competition with nearby plants, and survival, affect plant response and feedback.^2^ Plants use different strategies to adapt to environmental conditions, such as developing their root systems.^3^ ^,^ ^4^ Roots function to anchor the plant, absorb water and nutrients, and facilitate essential chemical communication with soil bacteria, other plants, and herbivores.^5^ ^,^ ^6^ This dynamic contact is primarily facilitated by root exudates, substances released by plant roots into the rhizosphere.^7^

Root exudates include a variety of inorganic and organic compounds that plants release into the soil,^8^ including primary metabolites like sugars, amino acids, and organic acids, as well as secondary metabolites.^9^ ^,^ ^10^ The composition and quantity of root exudates vary depending on plant species, developmental stage, and environmental factors.^11^ Through these exudates, plants can influence soil qualities, shape microbial communities, and regulate interactions with other organisms.^12^ They play a crucial role in nutrient acquisition by modifying the rhizosphere, enhancing nutrient solubility, and fostering symbiotic relationships with beneficial microorganisms, such as mycorrhizal fungi and nitrogen-fixing bacteria.^13^ Furthermore, root exudates can function as allelochemicals, promoting either competition or mutualism.^14^ Understanding root exudate occurrence is crucial in agricultural systems, where plant combinations can be optimized to increase productivity and reduce reliance on chemical fertilizers and pesticides.

Lupins (*Lupinus angustifolius* L.) belong to the Fabaceae family, commonly known as the legume family.^15^ They are well known for fixing atmospheric nitrogen through symbiotic relationships with rhizobia bacteria, making them valuable in sustainable farming systems.^16^ They are also cultivated for their protein-rich seeds, green manure, and soil-improving properties. Lupins also play a crucial role in enhancing soil fertility and structure due to their deep root systems and efficient nutrient uptake.^17^ Root exudates significantly influence the growth and function of lupin plants,^18^ and therefore, lupin plants exude specific compounds to enhance soil fertility,^19^ improve nutrient uptake,^20^ and shape the microbial community.^21^ However, lupin-derived alkaloids in the soil raise ecotoxicological risks and environmental concerns.^22^ ^,^ ^23^

Previous field studies have detected alkaloids and flavonoids associated with *Lupinus angustifolius*, indicating that these compounds can enter the surrounding environment. For example,^24^ detected apigenin, genistein, luteolin, and rutin in soil collected from *L. angustifolius* field plots, and alkaloids, including gramine, hydroxylupanine, lupanine, lupinine, and sparteine, were reported in lupin plant tissues and environmental samples.^22^ ^,^ ^23^ While lupin roots are known to substantially alter their rhizosphere environment, little is known about their ecological roles and the range of compounds they exude, particularly secondary metabolites such as alkaloids and flavonoids. The hypothesis is that lupin roots exude certain alkaloids and flavonoids for their ecological roles. Therefore, this study aims to test this hypothesis and investigate the chemical profile of lupin root exudates during the growing season using targeted metabolomics. The objectives are to (a) establish a root exudate high-throughput collection platform, (b) characterize the composition of specific alkaloids and flavonoids of lupin root exudates, and (c) profile the dynamic changes over time. Previous studies provided the basis for selecting the target compounds in the present study, representing environmentally relevant metabolites rather than a comprehensive representation of the alkaloid and flavonoid profiles of *L. angustifolius*.^22-24^ By investigating these metabolites in root collection solutions, we aimed to characterize their occurrence under controlled collection conditions. This knowledge and methodological framework provide a basis for future studies investigating how lupin biochemical traits may influence ecological interactions.

## Materials and methods

### Plant cultivation

This experiment was designed to develop protocols for collecting alkaloid and flavonoid root exudates from lupin roots by testing different collection media and times, and to profile and quantify root exudation during the growing season.

Seeds of blue lupin (*L. angustifolius*, cv. Borweta, cv. Mirabor, and cv. Bordaka) were planted in pots filled with sandy clay soil. While three cultivars were initially evaluated to confirm the consistency of the extraction protocol, this study focused on cv. Borweta (referred to hereafter as the primary cultivar) to provide a high-resolution temporal profile of exudation. To ensure the representativeness of our findings, the cultivars (cv. Mirabor and cv. Bordaka) were grown under identical conditions; growth and exudation data for these cultivars are provided in the Supplementary Information (Table S1) and show no significant deviations from the primary results. Cultivar Borweta is an indeterminate, branching cultivar known for its robust vegetative growth and high yield stability under varying environmental conditions. The Cultivar Mirabor is an early-maturing cultivar with an indeterminate growth habit, selected for its improved resistance to anthracnose and high protein efficiency. Cultivar Bordaka is a semi-determinate cultivar characterized by a more compact architecture and synchronized ripening.^25^ The pots were placed in greenhouse conditions. Drip irrigation was used throughout the experiment to ensure a sufficient water supply for growing lupin plants. To establish the root exudate collection protocol, lupin seeds were grown for 20 d. These lupin plants were used to test which collection medium and collection period best extract alkaloids and flavonoids from lupin root exudates. The protocol was later used to collect alkaloids and flavonoid root exudates during the growing season. To characterize the temporal dynamics of root exudation, samples were collected at five distinct developmental stages: early vegetative (20 d after sowing (DAS)), late vegetative (30 DAS), flowering/anthesis (60 DAS), pod filling (90 DAS), and physiological maturity (110 DAS). This sampling schedule was designed to capture the physiological shift during vegetative growth. By 110 DAS, the plants had completed pod development, allowing for a comprehensive assessment of how alkaloid and flavonoid exudation profiles evolve as the plant transitions into reproductive senescence.

### Collection of root exudates and determination of plant parameters

To collect root exudates from lupin roots, the pot with the lupin plant was moved to the lab, and the entire root system of each intact plant was carefully washed to remove the soil. Root exudate metabolite profiling was conducted using three biological replicates (*n* = 3) to provide an overview of the targeted alkaloid and flavonoid profiles under the different collection conditions. To assess the effect of the collection medium on root exudation, the plants were transferred to 1 L Erlenmeyer flasks (one plant per flask) (Figure 1), which were filled with 1 L either deionized water or 0.01 and/or 0.05  mM CaCl_2_. Researchers have used dilute CaCl_2_ solutions in root exudate collection protocols to provide a defined ionic environment during sampling.^26^ ^,^ ^27^ The concentrations used here were compared to assess their suitability for recovering lupin root-exuded alkaloids and flavonoids. The flasks were wrapped in aluminum foil to maintain darkness around the roots while keeping the stems and leaves above the waterline, and the collection experiment was conducted under the same controlled climate conditions as during the plant growth phase. To remove exudates from potentially damaged cells due to root washing, the roots were initially placed in the solution (water or CaCl_2_) for 10 min. The solutions obtained during the 10 min were discarded and not analyzed. Subsequently, fresh collection solutions were added, and the plants were allowed to exude into the solution (water or CaCl_2_) to evaluate the effect of the collection medium. Blank samples were prepared by filling a clean flask with either water or a CaCl_2_ solution, then leaving it in the greenhouse and treating it similarly to the exudate samples. Once the root exudation was collected, the roots were harvested and oven-dried for 72 h at 70 °C to determine the root biomass. The root biomass was measured using six biological replicates (*n* = 6) to provide enough replication for root phenotyping, as biomass sampling was destructive; plant allocation was planned to accommodate this measurement. The root lengths were measured manually.

**Figure 1.**
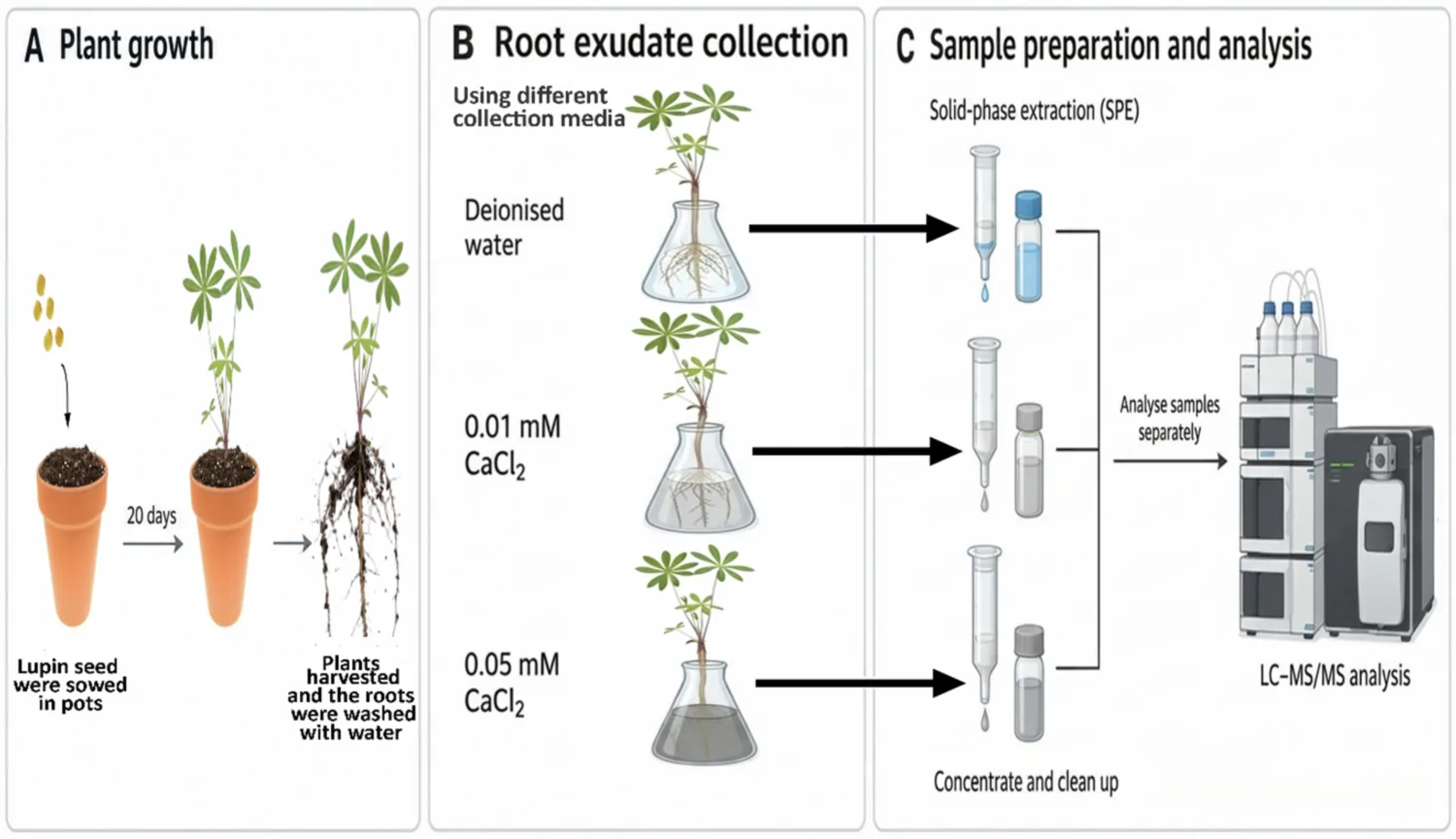
Schematic representation of the root exudate collection procedure and sample preparation for LC–MS/MS analysis.

### Time course experiment

To evaluate the effect of collection duration on the concentration of root exudates, 20-day-old lupin plants were used at six collection time points: 3, 6, 12, 24, 48, and 72 h. Each collection duration was evaluated using a separate set of plants, with one plant transferred to each 1 L Erlenmeyer flask containing 1 L of deionized water. The collection solution remained in contact with the roots throughout the assigned collection period and was collected only at the end of that period. Thus, the time points represented independent collection periods rather than successive sampling or solution replacement using the same plants. Once the root exudate collection time course was defined, additional lupin plants were used to investigate the profile and quantity of root exudates during the growing season at 20, 30, 60, 90, and 110 DAS.

### Processing exudate solutions

The root exudates were processed using the method described previously.^22^ ^,^ ^23^ Briefly, aliquots of the collected exudate solutions were filtered through filter paper (Whatman® Quantitative-Grade 5) and acidified to pH 3 with 0.01 mM HCl. The solutions were passed through an Oasis MCX solid-phase extraction (SPE) cartridge (6 cm^3^, 150 mg; Waters, Milford, Massachusetts), preconditioned sequentially with 5 mL MeOH and 5 mL Milli-Q water at 10 mL/min. The SPE cartridges were eluted with 5 mL MeOH, followed by 10 mL aqueous ammonia (2%, v/v), and the eluate was evaporated to dryness under a gentle N_2_ flow, then reconstituted in 1.0 mL 10% MeCN (v/v). The solution was filtered through a 0.22 μm PTFE syringe filter before LC–MS/MS analysis.

### LC–MS/MS analysis

The targeted analysis of a liquid chromatography-mass spectrometry (LC–MS/MS) system was used to analyze five alkaloids (13-hydroxylupanine, gramine, lupinine, lupanine, and sparteine) and four flavonoids (apigenin, genistein, luteone, and rutin) in lupin root exudate. These compounds were selected based on previous field studies reporting their occurrence in lupin-associated soil and other environmental samples, making them relevant targets analysis.^22-24^ ^,^ ^28^ The targeted analysis was for nine metabolites and was not intended to provide a comprehensive profile of all metabolites released by lupin roots.

An Agilent 1290 LC system equipped with an Agilent 6460 triple quadrupole mass spectrometer with a Jetstream electrospray source was used in positive ion mode for detection. Zorbax Eclipse Plus C-18 columns (2.1 mm × 50 mm × 1.8 μm) were used for chromatographic separation. Standard and root exudate solutions were injected into a pre-heated column at 35 °C, with a controlled flow rate of 0.4  mL/min. The mobile phases of eluent A (10% acetonitrile in water +0.1% formic acid, v/v) and eluent B (90% acetonitrile in water +0.1% formic acid, v/v) were used in gradient elution mode: 0.5–1 min 95% A, 1–2 min decreasing to 90% A, 3–10 min decreasing to 40% A, 11–14 min decreasing to 10% A, 14–16.5 min increasing to 95% A, and 16.5–24 min equilibrating at 95% A.

The mass spectrometer was operated in positive and negative ion mode with the following parameters: nitrogen drying gas temperature 350 °C, nitrogen sheath gas temperature 400 °C, nitrogen drying gas flow 10 L/min, nitrogen sheath gas flow 11 L/min, nebulizer pressure 55 psi, capillary voltage 5000 V and nozzle voltage 1000 V. During LC–MS/MS analysis, multiple reaction monitoring (MRM) transitions were used to ensure both the quantification and structural confirmation of alkaloid and flavonoid compounds (Table 1). One multiple reaction monitoring (MRM) transition served as a quantifier transition, and a second MRM transition served as a qualifier transition. Calibration curves were developed using standard solutions of alkaloids (13-hydroxylupanine, lupinine, lupanine, sparteine, and gramine) and flavonoids (genistein, apigenin, luteone, and rutin) ranging from 0.5 to 256 μg/L, enabling accurate quantification; dilution adjustments were made for samples exceeding the calibration range. To ensure reliability, analytical standards and instrumental blanks were regularly analyzed, confirming the absence of alkaloid and flavonoid contamination. To monitor instrumental response and potential drifts in the MS/MS system, alkaloid and flavonoid analytical standard solutions of 25 ng/L were analyzed after every 10 injections. Additionally, at least one instrumental blank sample was included in every set of 10 injections, and no alkaloids and flavonoids were present in the blanks.

**Table 1.** Compound-dependent parameters and multiple reaction monitoring (MRM) transitions were used to quantify alkaloids and flavonoids by LC–MS/MS.

Compound name	Chemical formula	Molecular weight (g/mol)	Precursor ion (m/z)	Fragments (m/z)	Collision energy (V)
13-Hydroxylupanine	C_15_H_24_N_2_O_2_	264.369	265.1911	152.107	26
Lupinine	C_10_H_19_NO	169.268	170.1539	124.112	24
Lupanine	C_15_H_24_N_2_O	248.370	249.1961	136.112	25
Sparteine	C_15_H_26_N_2_	234.387	235.2169	134.097	27
Gramine	C_11_H_14_N_2_	174.24	175.01	130.067	12
Genistein	C_15_H_10_O_5_	270.24	271.060	223.062	−10
Apigenin	C_15_H_10_O_5_	270.24	271.060	158.012	−15
Luteone	C_20_H_18_O_6_	354.4	352.88	200.880	−40
Rutin	C_27_H_30_O_16_	610.5	609.57	301.100	−50

### Data and statistical analysis

To account for differences in plant size across the five developmental stages, root exudation was expressed per dried root weight. The exudation rate was then calculated as the individual concentration of detected alkaloids or flavonoids per unit root dry weight (DW) biomass, expressed as micrograms per gram of root DW. The pots in the greenhouse unit were organized in a Randomized Complete Block Design with three replicates. A one-way ANOVA was conducted using XLSTAT statistical software (version 2019.2.2) to assess whether there were significant differences between the solutions used to collect the root exudates and to evaluate the impact of collection media on alkaloid and flavonoid concentrations at each sampling interval. Tukey’s HSD test (*p ≤ 0.05*) was applied for mean comparisons.

## Results

This study optimized the protocol for collecting root exudate alkaloids and flavonoids from lupin roots and quantified their concentrations throughout the growing season.

The temporal progression of fresh (green squares) and dry (yellow circles) root biomass (g) of lupin plants observed over a growth period from 10 to 110 DAS is shown in Figure 2. Fresh root biomass remained low and stable up to 45 DAS, then increased rapidly between 60 and 90 DAS, peaking at approximately 100 g before slightly declining by 110 DAS. Additionally, dry root biomass gradually increased over the study period, reaching a maximum of approximately 25 g at 110 DAS. This trend highlights the dynamic changes in root development and water content during the lupin growth cycle.

**Figure 2.**
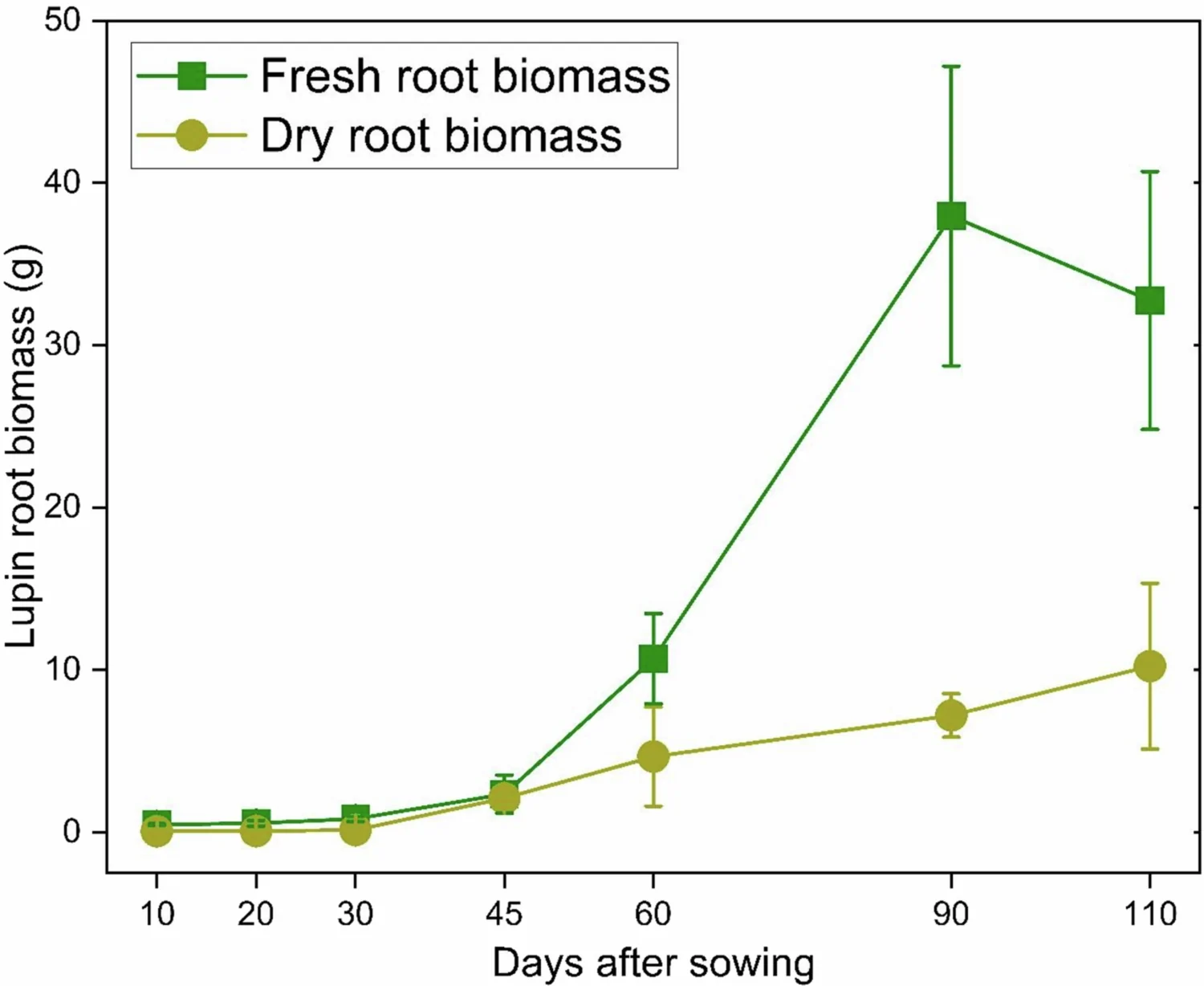
Fresh and dry biomass of lupin plants (grams) was assessed at intervals between 10 and 110 d after sowing. Plants were randomly selected from the greenhouse for analysis. Data are presented as means (*n* = 6), with error bars indicating standard deviations.

Root length increased progressively from 10 to 110 DAS (Figure 3). The initial growth was slow between 10 and 20 DAS, followed by rapid elongation between 20 and 60 DAS, when the root length increased from approximately 4 cm to nearly 50 cm. Subsequently, root length reached over 60 cm by 110 DAS. This pattern reflects typical early-phase expansion followed by a plateau, likely because the plant shifted its resource allocation from root extension to biomass accumulation and maturation.

**Figure 3.**
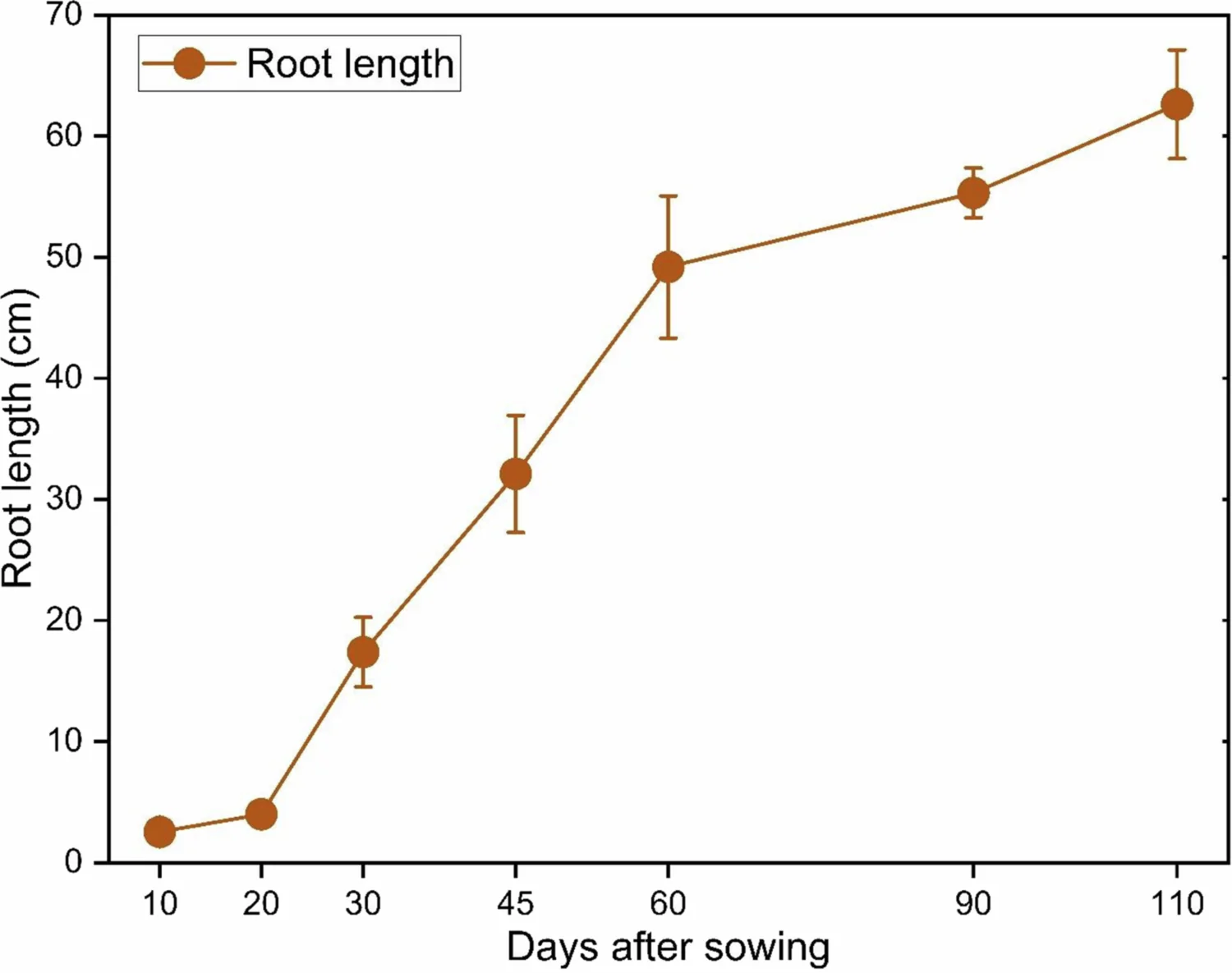
The root length of lupin plants (centimeters) was assessed at intervals between 10 and 110 d after sowing. Root length measurements were performed on the same plants collected for biomass determination. Data are presented as means (*n* = 6), with error bars indicating standard deviations.

To evaluate the effect of collection-medium composition on the recovery of alkaloid and flavonoid concentrations was compared from 20-day-old lupin plants using deionized water, 0.01  mM CaCl_2_, and 0.05 mM CaCl_2_ as collection media (Figure 4). The results indicated that deionized water was more effective than CaCl_2_ as the collection medium for collecting higher concentrations of root exudates, alkaloids, and flavonoids. The concentrations of 13-hydroxylupanine, gramine, lupanine, lupinine, and sparteine were substantially higher in water than in both CaCl₂ treatments, but lupinine and sparteine were significantly higher in the deionized water medium (one-way ANOVA, *P < 0.009* and *0.002*, respectively). The same pattern was observed for flavonoids, and the rutin concentration was significantly higher in the deionized water medium (one-way ANOVA, *P < 0.009*).

These results suggest that the collection medium influences the composition and concentration of root exudates, with deionized water favoring higher exudation of both alkaloids and flavonoids than calcium-containing solutions. Higher exudate collection in deionized water suggests that ionic strength or calcium ions may inhibit their release or stability.

**Figure 4.**
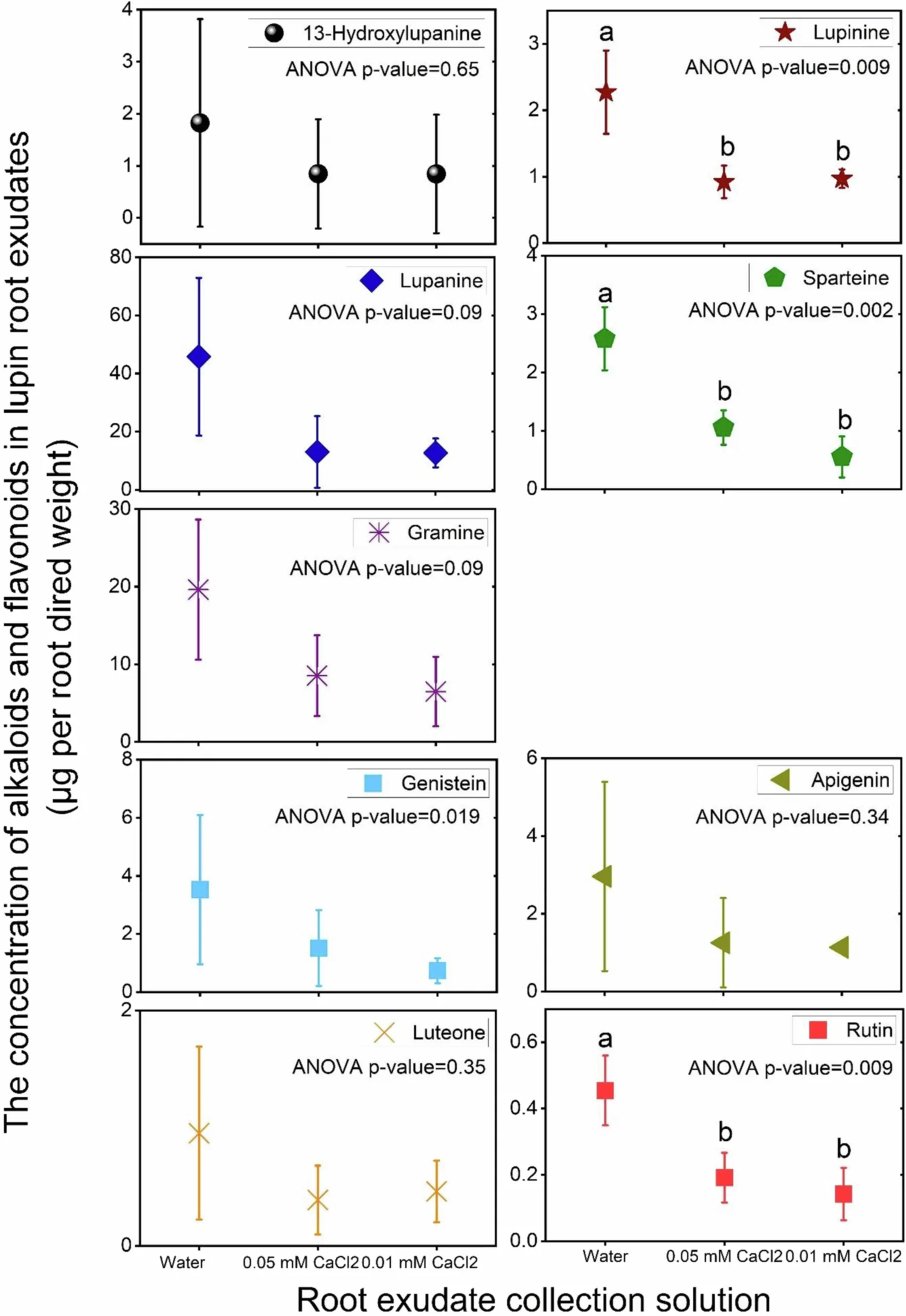
Root exudate collection of alkaloids and flavonoids from 20-day-old lupin roots using either deionized water or 0.01 or/and 0.05 mM CaCl_2_ as collection media. Data are presented as mean concentrations of alkaloids and flavonoids (*n* = 3), with error bars representing standard deviations.

The temporal dynamics of exuded alkaloid concentrations in lupin root exudates were observed over a range of collection times (3–72 h), specifically at 3, 6, 12, 24, 48, and 72 h (Figure 5). Among the analyzed alkaloids, lupanine and gramine were detected at comparatively higher concentrations than the other compounds throughout the sampling period. Sparteine tended to accumulate more at 24 h, whereas 13-hydroxylupanine and lupinine showed more moderate fluctuations over time. The concentration of alkaloids: 13-hydroxylupanine, gramine, lupinine, lupanine, and sparteine, showed minimal exudation during the initial 3-h collection. Overall, alkaloids accumulated during the early-to-intermediate sampling intervals (12–24 h), then stabilized or declined at later time points (48–72 h). The analysis of variance (one-way ANOVA) showed no significant changes over time. However, this trend suggests that maximum alkaloid exudation occurs within the first 24 h, after which release diminishes. Alkaloid release from lupin roots varies over time, underscoring the importance of collection time when assessing root exudation profiles.

**Figure 5.**
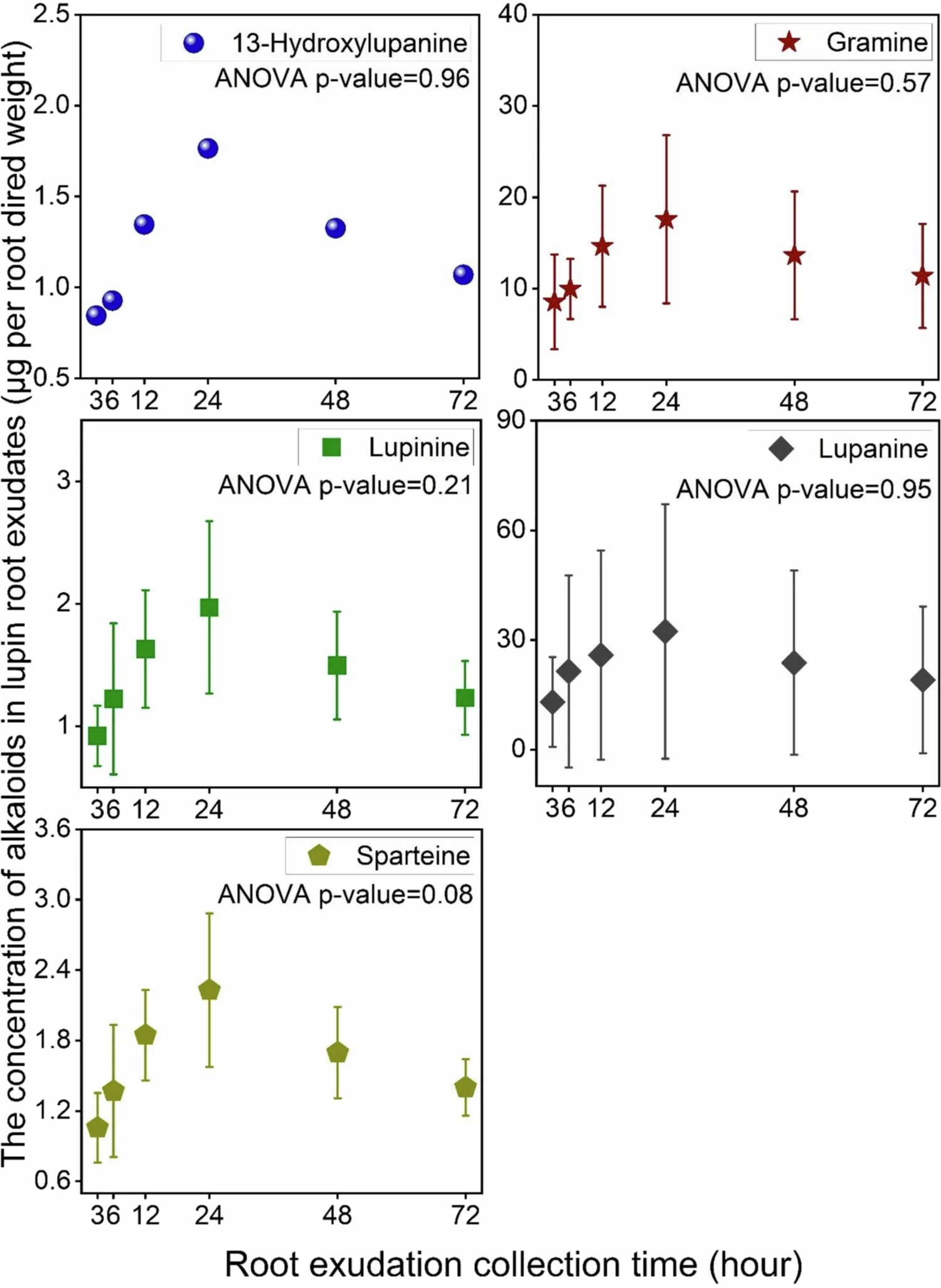
Temporal dynamics of alkaloid exudation from 20-day-old lupin (*Lupinus* spp.) roots were collected over a 3–72 h sampling period using deionized water as the exudate collection medium. Five alkaloids were detected in root exudates, including 13-hydroxylupanine, gramine, lupinine, lupanine, and sparteine. Alkaloid concentrations are expressed as μg g^−1^ root dry weight and presented as mean values (*n* = 3), with error bars indicating standard deviations. One-way ANOVA showed that collection time did not significantly affect exudation levels of individual alkaloids under the tested conditions (*p > 0.05*).

Flavonoid levels exuded by 20-day-old lupin roots were measured in deionized water over a range of collection times (3–72 h) (Figure 6). The temporal dynamics of exuded flavonoid concentrations in lupin root exudates were similar to those of alkaloids. The highest exudation levels of flavonoids, apigenin and genistein, luteone, and rutin were observed at 24 h. In contrast, the lowest flavonoid exudation was recorded at first 3 h. Flavonoid release increased progressively, reaching a maximum at 24 h, after which it declined as the incubation time extended to 72 h. Flavonoid exudation in lupin roots peaks at 24 h, likely due to active metabolism. Rutin concentrations changed significantly over time, with concentrations during the first 12 h higher than at later collection times. The decline of flavonoid concentrations after 24 h may result from resource depletion, reduced exudation after an initial burst, and enzymatic or microbial degradation in solution. Thus, emphasizing the importance of optimal collection timing.

**Figure 6.**
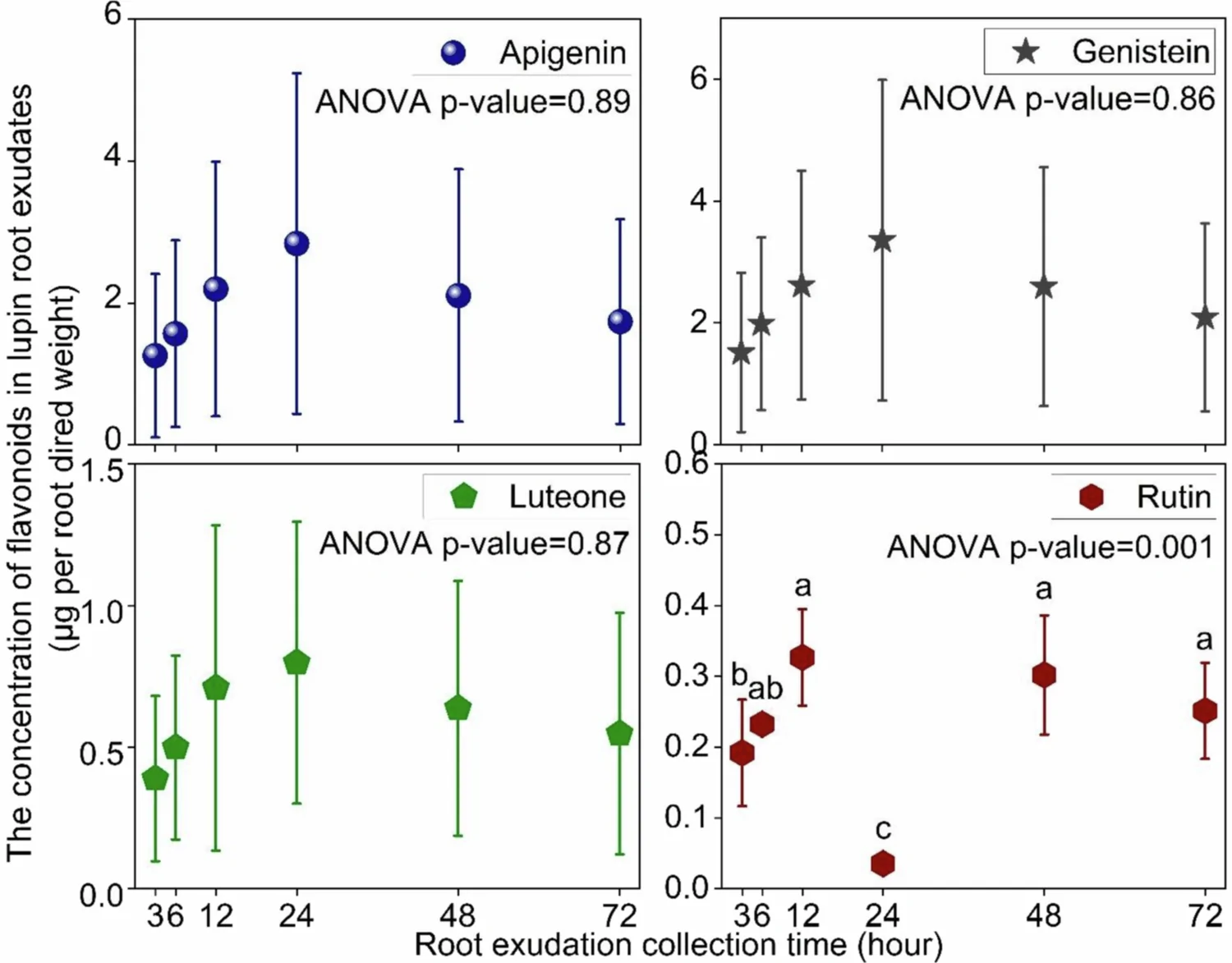
Temporal dynamics of flavonoid exudation from 20-day-old lupin (*Lupinus* spp.) roots were collected over a 3–72 h sampling period using deionized water as the exudate collection medium. Four flavonoids were detected in root exudates, including apigenin, genistein, luteone, and rutin. Alkaloid concentrations are expressed as μg g^−1^ root dry weight and presented as mean values (*n* = 3), with error bars indicating standard deviations. One-way ANOVA showed that collection time did not significantly affect the exudation levels of individual alkaloids under the tested conditions (*p > 0.05*).

The developmental dynamics of alkaloid exudation from lupin roots were assessed at various time points (10–110 DAS) during the growing season (Figure 7). Alkaloid concentrations varied significantly across plant developmental stages, demonstrating strong temporal regulation of alkaloid exudation during lupin growth. In particular, gramine, lupinine, and sparteine decreased significantly with plant age (ANOVA *p-values = 0.001*, *0.006*, and *0.001*, respectively), with the highest exudation at early vegetative stages (20 DAS), followed by marked reductions at later stages (60–110 DAS). Gramine concentrations decreased sharply from approximately 16 μg g^−1^ root dry weight at 20 DAS to nearly undetectable levels after 60 DAS (ANOVA, *p = 0.001*). Similarly, sparteine exudation declined progressively throughout plant development, with significantly higher concentrations detected at 20 DAS than at later stages (ANOVA, *p = 0.001*). Lupinine also showed a significant developmental-stage effect (ANOVA, *p = 0.006*), although concentrations at 30 DAS remained intermediate and not significantly different from 20 DAS. In contrast, 13-hydroxylupanine and lupanine did not vary significantly across developmental stages (ANOVA *p-values = 0.15* and *0.12*, respectively), despite relatively high variability during early growth stages. Among all compounds analyzed, lupanine exhibited the highest variability, particularly at 20 DAS. Expressing alkaloid concentrations relative to the volume of the collected root exudate solution (μg L^−1^) revealed a temporal pattern similar to that observed for the biomass-normalized data, with the highest alkaloid concentrations detected during the early developmental stages, followed by a progressive decline as plant growth advanced (Figure S1). The same alkaloid dynamic patterns were observed in other tested genotypes (Table S1). These findings indicate that lupin root alkaloid exudation is developmentally regulated, with the greatest release occurring during early growth stages, suggesting that young plants may allocate more secondary metabolites to rhizosphere interactions and defense-related functions during early establishment.

**Figure 7.**
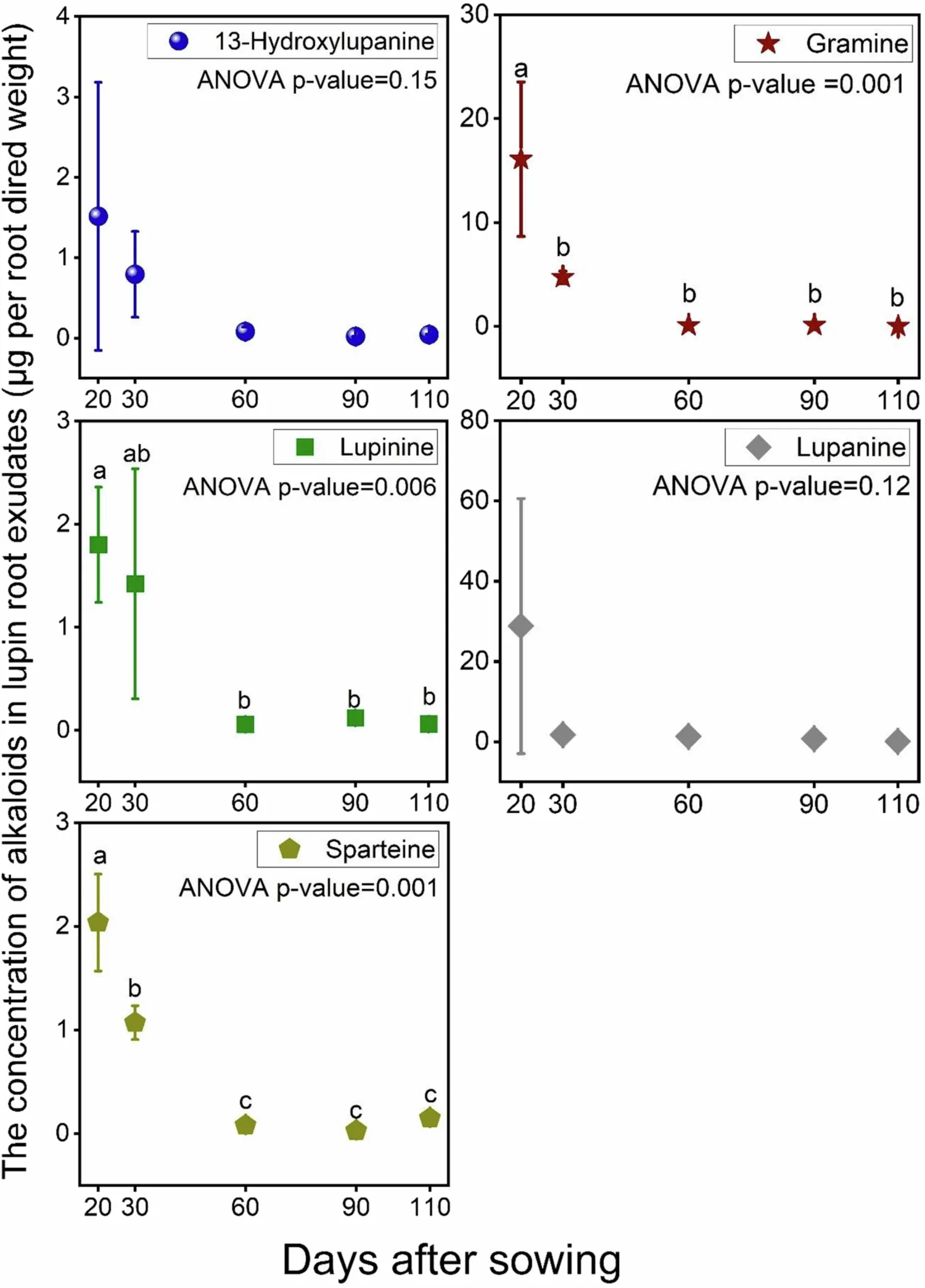
Seasonal dynamics of alkaloid exudation from lupin (*Lupinus* spp.) roots were collected at different developmental stages ranging from 20 to 110 d after sowing (DAS). Alkaloid concentrations detected in root exudates, including 13-hydroxylupanine, gramine, lupinine, lupanine, and sparteine, are expressed as μg g^−1^ root dry weight and shown as mean values (*n* = 3), with error bars representing standard deviations. Different lowercase letters indicate significant differences among developmental stages according to Tukey’s post hoc test following one-way ANOVA (*p < 0.05*).

The developmental profiles of flavonoid exudation from lupin roots showed variation across stages at 20, 30, 60, 90, and 110 DAS (Figure 8). Flavonoid exudation decreased significantly with plant age (one-way ANOVA *p-values = 0.05*), with the highest exudation at the early vegetative stages (20 DAS), followed by marked reductions at later stages (60–110 DAS). Apigenin, genistein, and luteone were exuded at low concentrations at 20–30 DAS, then increased progressively, peaking at 110 DAS. Low exudation in the early stages, but an increase at 110 DAS, suggesting it is a late-stage metabolite, with a possible increase during vegetative growth. The significant rise suggests a role in the reproductive phase or in rhizosphere interactions during seed production. Rutin increased at 30 DAS, then declined, reaching a minimum at 90 DAS, followed by a slight rebound at 110 DAS. The results highlight the dynamic nature of flavonoid exudation, which varies with growth stages. The increased accumulation of these flavonoids at the final sampling point indicates heightened metabolic activity and secondary metabolite production as the plant develops. When flavonoid concentrations were expressed relative to the volume of collected root exudate solution (μg L^−1^), a comparable developmental trend emerged, with the greatest flavonoid accumulation occurring during early growth stages and gradually decreasing as the plants matured (Figure S2). The same flavonoid dynamics were observed for the other tested genotypes (Table S2). Understanding these exudation patterns provides insights into the physiological role of flavonoids in plant development and their potential applications in soil–microbe interactions and plant resilience.

**Figure 8.**
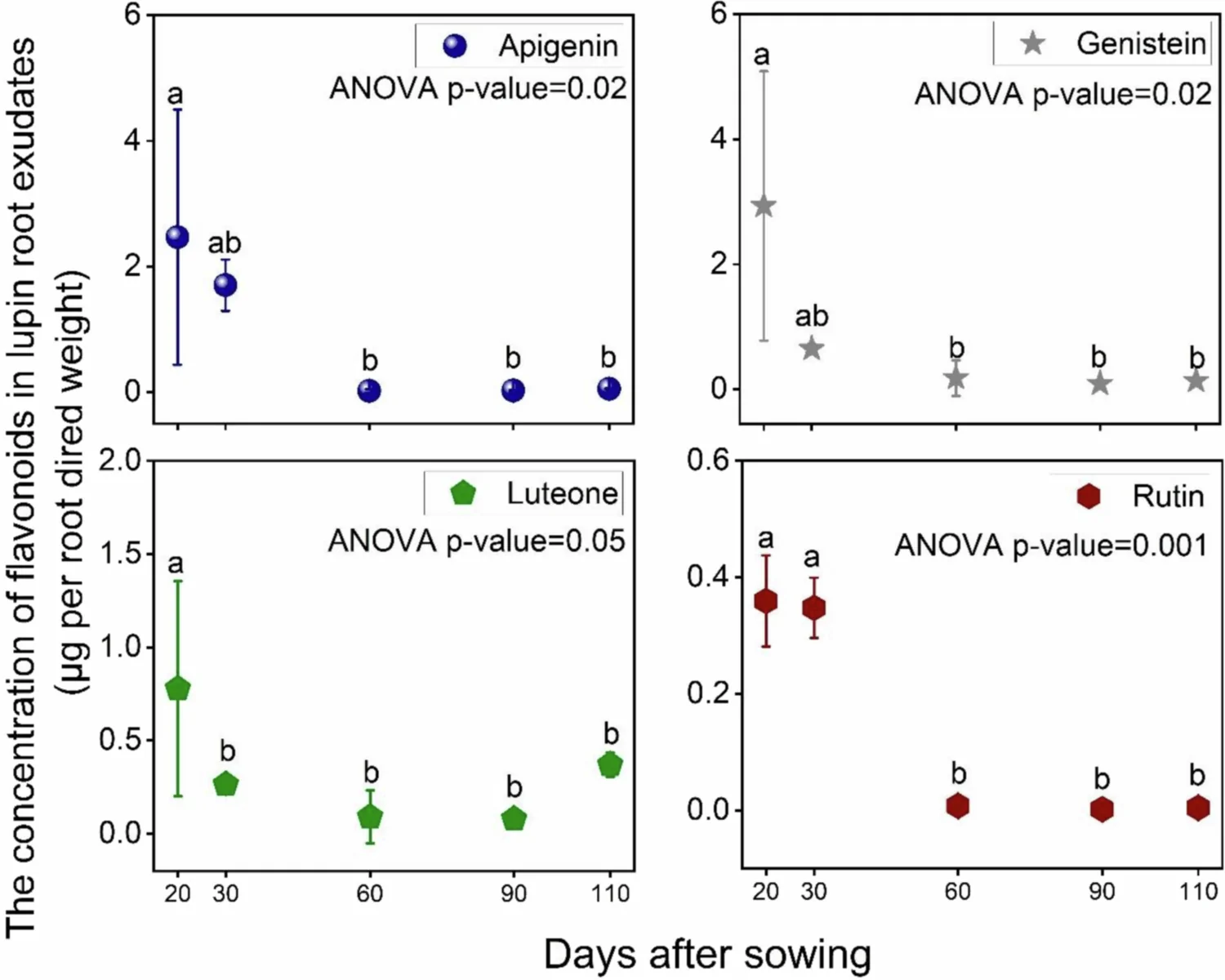
Seasonal dynamics of flavonoid exudation from lupin (*Lupinus* spp.) roots collected at different developmental stages ranging from 20 to 110 d after sowing (DAS). Flavonoid concentrations detected in root exudates, including apigenin, genistein, luteone, and rutin, are expressed as μg g^−1^ root dry weight and shown as mean values (*n* = 3), with error bars representing standard deviations. Different lowercase letters indicate significant differences among developmental stages according to Tukey’s post hoc test following one-way ANOVA (*p < 0.05*).

## Discussion

This study examined how time intervals affect root exudate profiles, including alkaloids and flavonoids, in lupin plants. It also analyzed the impact of water and CaCl_2_ as collection media on root exudation to better understand their role in plant growth and lupin's biochemical interactions.

The study assessed lupin root biomass (fresh and dry weights) at intervals from 10 to 110 d after sowing to evaluate developmental patterns and physiological adaptations. Root biomass accumulation is influenced by nutrient availability, hormonal regulation, and root exudates.^10^ ^,^ ^29^ Fresh biomass reflects the current physiological state of the roots, while dry biomass provides insight into structural growth and long-term carbon distribution. These data indicate that fresh root biomass may be affected by environmental factors such as senescence or post-peak changes in resource allocation, while dry root biomass persisted, likely due to continued structural growth and lignification. Typically, root biomass grows over time, with a slow initial phase during establishment, followed by rapid accumulation during the vegetative stage, and then leveling off as reproductive development begins. Variations in biomass accumulation can be driven by environmental conditions and genetic differences among lupin species.^30^ The temporal patterns of root biomass development in lupin plants highlight the critical physiological and biochemical processes governing root growth and functionality.

Root progressive elongation suggests that root development is closely linked to plant growth stages, with an initial slow phase, a rapid expansion phase, and a later steady growth phase. Understanding the growth patterns is essential for optimizing cultivation practices and improving resource allocation for lupin production. Root length is a key factor in how plants acquire water and nutrients. The regular assessment of root length helps evaluate root system growth and its adaptive responses to changing environmental conditions.^31^ During the early stages, root elongation is crucial for exploring the soil. In the mid-stages, it is associated with improved resource absorption efficiency, and in the final stage, root length typically stabilizes as plants focus on developing reproductive structures. Root length provides insights into growth dynamics, allowing the identification of factors that promote or limit root expansion, such as soil compaction,^32^ nutrient availability,^33^ and microbial interactions.^34^

A fixed sampling volume of 1 L was used for all exudate collections to ensure consistency across treatments and developmental stages. The sampling volume can influence apparent exudation rates by affecting metabolite dilution and diffusion gradients. As highlighted by Otxandorena-Ieregi et al.,^35^ variations in solution volume can significantly impact exudate recovery and interpretation. While our experimental design maintained a constant volume to allow direct comparisons among treatments, future studies could further refine the root-to-solution ratios to better approximate rhizosphere conditions and improve cross-study comparability.

The collection medium influenced the concentrations of alkaloids and flavonoids recovered from the roots of 20-day-old lupin plants. For all targeted compounds, deionized water yielded higher concentrations than either 0.01 or 0.05 mM CaCl_2_. This finding indicates that the physicochemical properties of the collection medium affect the recovery of root-exuded metabolites. Under the conditions tested, deionized water was therefore a suitable collection medium for the targeted analysis of lupin root-exuded alkaloids and flavonoids, although this finding does not establish it as the optimal medium for other plant species or classes of root exudates. Root exudation is a complex process affected by factors such as root membrane permeability, transporter activity, and environmental conditions.^36^ Quinolizidine alkaloids from lupins are nitrogen-containing secondary metabolites, primarily formed in the roots.^37^ They are exuded either through passive diffusion or active secretion. Higher exudation observed in deionized water may be due to the absence of ionic interactions that typically restrict alkaloid diffusion in saline solutions such as CaCl_2_. Similarly, flavonoids, synthesized from the phenylpropanoid pathway,^38^ are released via proton-dependent multidrug.^39^ The presence of Ca^2+^ ions in the collection medium can disrupt this pH-dependent mechanism, reducing flavonoid secretion compared to deionized water. The data demonstrate that water is an effective solution for collecting a broad spectrum of alkaloids and flavonoids from lupin root exudates. The significant reductions in metabolite concentrations with CaCl_2_ solutions should be considered when designing experiments, especially those aimed at profiling secondary metabolites or studying rhizosphere interactions. Deionized water, acting as a neutral medium, preserves root membrane integrity and allows for passive diffusion. In contrast, CaCl_2_ solutions containing Ca^2+^ ions may alter membrane permeability, disrupt transporter function, and form ionic interactions with negatively charged flavonoids, reducing their solubility and diffusion.^40^ Furthermore, Ca^2+^ stabilizes the cell wall, potentially limiting exudate release. Thus, research studies on root exudates for chemical profiling, allelopathy, or plant–microbe interactions should standardize the collection medium, ideally by testing different compositions and ionic strengths.

We evaluated the effect of collection duration on the alkaloid and flavonoid concentrations recovered from 20-day-old lupin roots at 3, 6, 12, 24, 48, and 72 h (Figures 5 and 6). Root exudation profiles of lupin reveal compound-specific and time-dependent responses to collection conditions. Both alkaloids and flavonoids peak around 24 h, suggesting a coordinated exudation profile across chemical classes. However, these measurements reflect amounts recovered from the collection solutions at the respective endpoints and do not independently distinguish changes in metabolite release from potential degradation, re-uptake, or other processes during collection. This finding has implications for experimental design, rhizosphere interaction studies, and metabolomic profiling. Differences among collection durations may reflect changes in metabolite release from roots, but they may also be influenced by processes occurring after release, including microbial degradation, chemical instability, and re-uptake by roots. These processes were not directly measured in this study, and their relative contributions therefore remain uncertain. Consequently, the observed concentration patterns should not be interpreted as direct evidence of changes in biological secretion rates or as proof that metabolite release peaked at a particular time. Rather, the results identify how collection duration affected the recovery of the targeted metabolites under the experimental conditions used. Future experiments incorporating microbial controls, compound-stability tests, and shorter-interval measurements could help distinguish metabolite release from subsequent losses in the collection solution.

The exudation of alkaloids (13-hydroxylupanine, gramine, lupinine, lupanine, and sparteine) and flavonoids (apigenin, genistein, luteone, and rutin) was measured for 3–72 h after collecting root exudates from 20-day-old lupin plants using deionized water. Time-course analyses showed that both alkaloid and flavonoid exudation peaked at approximately 24 h of collection, suggesting that active secretion is likely facilitated by membrane-bound transporters or passive diffusion across root epidermal cells. The biosynthetic pathways of alkaloids and flavonoids might be influenced by enzymatic activity and environmental factors. The exudation of these compounds plays important ecological roles, including plant defense, microbial interactions, and allelopathy, all of which affect rhizosphere dynamics.^41^ Variations in exudation rates may also reflect changes in root metabolic activity, cellular permeability, or microbial degradation in the surrounding medium.^42^ These results offer important insights into the timing and dynamics of bioactive metabolite secretion and its potential role in plant‒microbe interactions. These results underscore the importance of a 24-h sampling window to capture peak exudation from roots and avoid under- or over-estimation due to early collection or compound degradation over time.

Root exudates of lupin plants were collected at intervals from 20 to 110 DAS to measure the temporal release of these compounds. Exudation varied significantly over the growing period, suggesting that the plant’s physiological development and metabolic shifts influence the production and secretion of these compounds. Alkaloids are often involved in plant defense against herbivores and pathogens,^43^ while flavonoids contribute to plant protection through their antioxidant and antimicrobial properties.^44^ Some compounds (e.g., gramine) are exuded early, potentially aiding seedling establishment and protection, while others (e.g., sparteine, lupinine) are exuded later, likely supporting long-term defense or signaling during reproduction. Flavonoid exudation is also developmentally regulated, with compound-specific timing. Luteone and genistein show strong late-stage accumulation, while rutin is dominant in the early stages. The variation in exudation levels across intervals may be attributed to changes in root metabolic activity, enzyme expression, and root cell membrane permeability over time. Additionally, interactions with rhizosphere microbiota could influence exudate release, as root-associated microbes may either degrade or stimulate the production of certain exudates.^10^

An important limitation of the root exudate collection method is the potential contribution of compounds released from mechanically damaged root tissues. Although the roots were carefully washed and the initial wash solution was discarded to reduce compounds released during handling, this procedure cannot completely exclude leakage from damaged root hairs or other root cells during subsequent collection. The present results are the concentrations of alkaloids and flavonoids recovered from the root collection solutions rather than regulated exudation rates. Future studies could use non-destructive approaches, such as rhizosphere perfusion or rhizodeposition traps, to reduce root disturbance and investigate metabolite release under conditions that better preserve root integrity.

## Conclusions

This study investigated how root exudate collection media, sampling duration, and plant developmental stage influence the release of alkaloids and flavonoids from *Lupinus angustifolius* roots. The results demonstrated that both the collection medium and sampling duration significantly affected the detected concentrations of secondary metabolites in the root exudates. Deionized water consistently yielded higher recoveries of alkaloids and flavonoids than CaCl_2_ solutions, indicating that collection conditions strongly influence exudate recovery and metabolite detection. Temporal analyses further revealed that metabolite accumulation in root exudates was most pronounced during the first 24 h of collection, highlighting the dynamic nature of root exudation processes. In addition, the developmental stage significantly influenced exudation patterns, with the highest alkaloid and flavonoid concentrations generally detected during early plant growth stages, followed by a gradual decline as plants matured. Similar developmental trends were observed when metabolite concentrations were expressed relative to root dry weight and exudate solution volume, demonstrating that developmental regulation strongly influences exudation profiles.

Importantly, this study focused on quantifying metabolite concentrations recovered from exudate collection systems rather than determining absolute in situ exudation rates in the rhizosphere. Therefore, the findings should be interpreted as comparative assessments of exudate recovery under controlled experimental conditions. Nevertheless, the optimized sampling workflow and targeted LC–MS/MS MRM approach provide a robust methodological framework for future studies investigating lupin root chemistry and rhizosphere interactions. Overall, our findings advance the methodological understanding of root exudate sampling in lupin and lay the foundation for future ecological and physiological studies examining the functional roles of alkaloids and flavonoids in plant–soil interactions. Future studies should further investigate how irrigation regimes, water quality, and alternative collection conditions influence root biomass development and metabolite exudation dynamics. Refining these environmental and methodological factors may improve our understanding of the physiological regulation of root exudation and support the development of more efficient and reproducible sampling strategies. Integrating this knowledge into sustainable agricultural practices could improve crop resilience, resource-use efficiency, and the management of beneficial rhizosphere interactions.

## Supplementary Material

SI.docx SI.docx

## Data Availability

Data will be made available on request.
