# Supplementary material for: Root exudates of alkaloids and flavonoids from Lupinus angustifolius plants
Source: Plant Signal Behav. 2026 Nov 5;21(1):2760037. doi: 10.1080/15592324.2026.2760037 (PMC13757663; doi:10.1080/15592324.2026.2760037)
Supplement: Supplementary file 1 [file KPSB_A_2760037_SM0818.docx]

**Root exudates of alkaloids and flavonoids from *Lupinus angustifolius* plants**

Hawar Halshoy^a^, Shwana Ahmed Braim^b^*, and Jawameer R. Hama^c^

^a^Horticulture and Landscape Design Department, College of Agricultural Engineering Sciences, University of Sulaimani, Sulaymaniyah (46001), Kurdistan Region, Iraq. [hawar.hama@univsul.edu.iq](mailto:hawar.hama@univsul.edu.iq), [hawarhalsho@gmail.com](mailto:hawarhalsho@gmail.com), ORCID ID: 0000-0002-9842-6178

^b^Chemistry and Forensics Department, School of Science and Technology, Nottingham Trent University, Nottingham, UK. [shwana.braim@ntu.ac.uk](mailto:shwana.braim@ntu.ac.uk), [ORCID ID: 0000-0002-0019-8972](https://orcid.org/0000-0002-0019-8972)

^c^Department of Agroecology, Aarhus University, Forsøgsvej 1, 4200 Slagelse, Denmark. [jawameer@agro.au.dk](mailto:jawameer@agro.au.dk), ORCID ID: 0000-0002-0663-4093

*Corresponding author: [shwana.braim@ntu.ac.uk](mailto:shwana.braim@ntu.ac.uk)

**Figure S1. Root exudate collection of alkaloids (13-Hydroxylupanine, gramine, lupinine, lupanine, and sparteine) during the growing season from 20 to 110 days after sowing. Data are presented as mean alkaloid concentrations (n = 3), with error bars representing standard deviations.**

**Figure S2.** **Root exudate collection of flavonoids (apigenin, genistein, luteone, and rutin) during the growing season from 20 to 110 days after sowing. Data are presented as mean flavonoid concentrations (n = 3), with error bars representing standard deviations.**

**Table S1. Concentrations (µg . g^-1^) of alkaloids (13-Hydroxylupanine, gramine, lupinine, lupanine, and** **sparteine) in the root exudate of Mirabor and Bordaka genotypes during the growing season from 20 to 110 days after sowing. Data are presented as mean alkaloid concentrations (n = 3) ± standard deviation.**

| **Cultivars** | **Days after sowing** | **13-Hydroxylupanine** | **Lupinine** | **Lupanine** | **Sparteine** | **Gramine** |
| --- | --- | --- | --- | --- | --- | --- |
| **Mirabor** | **20** | 1.66±1.83 | 1.78±0.55 | 28.6±31.5 | 2.02±0.46 | 15.9±7.37 |
|  | **30** | 0.81±0.54 | 1.45±1.12 | 1.27±0.99 | 1.10±0.12 | 4.76±0.60 |
|  | **60** | 0.08±0.05 | 0.06±0.02 | 1.17±1.31 | 0.08±0.03 | 0.07±0.03 |
|  | **90** | 0.02±0.01 | 0.13±0.05 | 0.56±0.45 | 0.03±0.01 | 0.09±0.04 |
|  | **110** | 0.05±0.01 | 0.08±0.02 | 0.13±0.12 | 0.19±0.06 | 0.01±0.01 |
| **Bordaka** | **20** | 1.66±1.87 | 1.92±0.48 | 28.6±31.8 | 2.18±0.35 | 17.3±8.31 |
|  | **30** | 0.85±0.57 | 1.52±1.18 | 1.33±1.03 | 1.15±0.12 | 5.00±0.63 |
|  | **60** | 0.09±0.05 | 0.06±0.02 | 1.23±1.37 | 0.09±0.04 | 0.08±0.04 |
|  | **90** | 0.02±0.01 | 0.14±0.07 | 0.56±0.48 | 0.03±0.01 | 0.10±0.04 |
|  | **110** | 0.05±0.01 | 0.08±0.03 | 0.14±0.12 | 0.20±0.06 | 0.01±0.01 |

**Table S2. Concentrations (µg . g^-1^) of flavonoids (13-Hydroxylupanine, gramine, lupinine, lupanine, and sparteine) in the root exudate of Mirabor and Bordaka genotypes during the growing season from 20 to 110 days after sowing. Data are presented as mean alkaloid concentrations (n = 3) ± standard deviation.**

| **Cultivars** | **Days after sowing** | **Genistein** | **Apigenin** | **Luteone** | **Rutin** |
| --- | --- | --- | --- | --- | --- |
| **Mirabor** | **20** | 4.45±1.59 | 3.47±0.39 | 0.44±0.01 | 0.33±0.09 |
|  | **30** | 0.66±0.10 | 1.77±0.54 | 0.19±0.15 | 0.36±0.06 |
|  | **60** | 0.19±0.30 | 0.02±0.03 | 0.01±0.01 | 0.01±0.01 |
|  | **90** | 0.09±0.05 | 0.03±0.01 | 0.08±0.05 | 0.01±0.01 |
|  | **110** | 0.12±0.09 | 0.06±0.01 | 0.42±0.08 | 0.01±0.01 |
| **Bordaka** | **20** | 3.15±2.37 | 2.66±2.25 | 0.35±0.18 | 0.39±0.10 |
|  | **30** | 0.70±0.10 | 1.86±0.56 | 0.20±0.16 | 0.38±0.07 |
|  | **60** | 0.20±0.32 | 0.02±0.03 | 0.01±0.01 | 0.01±0.01 |
|  | **90** | 0.09±0.06 | 0.03±0.01 | 0.07±0.06 | 0.01±0.01 |
|  | **110** | 0.16±0.03 | 0.06±0.01 | 0.33±0.26 | 0.01±0.01 |
